# Clinical outcome of patients with COVID-19 Pneumonia treated with corticosteroids and colchicine in Colombia

**DOI:** 10.1186/s12941-021-00460-9

**Published:** 2021-09-14

**Authors:** Miguel Alejandro Pinzón, Doris Cardona Arango, Juan Felipe Betancur, Santiago Ortiz, Héctor Holguín, Carolina Arias Arias, Bernardo J. Muñoz Palacio, Michael Amarillo, Juan Felipe Llano, Pablo Montoya

**Affiliations:** 1 Infectious Diseases Department, Clínica Medellín Grupo Quirónsalud, Nueva Clínica Sagrado Corazón, Clínica Panamericana, Carrera 65B # 30-95, Medellín, Colombia; 2 CES University, Medellín, Colombia; 3 Internal Medicine Department, Clínica Medellín Grupo Quirónsalud, Salud SURA, Medellín, Colombia; 4 Department of Mathematical Sciences, Universidad EAFIT, Medellín, Colombia; 5 Clínica Medellín Grupo Quirónsalud, Medellín, Colombia; 6 Pulmonology Department, Clínica Medellín Grupo Quirónsalud, Medellín, Colombia; 7 Nueva Clínica Sagrado Corazón, Medellín, Colombia; 8 Clínica Medellín Grupo Quirónsalud, Medellín, Colombia; 9 Internal Medicine Department, Nueva Clínica Sagrado Corazón, Medellín, Colombia

**Keywords:** Coronavirus, COVID-19, Colchicine, Corticosteroids, Pneumonia

## Abstract

**Background:**

To date, there is no specific antiviral therapy for severe acute respiratory syndrome Coronavirus 2 (SARS-CoV-2) that causes Coronavirus disease 2019 (Covid-19). Since there is no specific therapy against SARS-CoV2, current efforts aim to prevent contagion through public health measures and develop a protective vaccine. While waiting for the latter, it is necessary to evaluate the drugs that at least, in initial studies, suggested some degree of utility in the management of Covid-19 or its complications. The main objective of the study was to describe the clinical manifestations and outcomes of patients with severe Covid-19 Pneumonia treated with corticosteroids and colchicine.

**Materials and methods:**

A cross sectional study of 301 adult patients with Covid-19 Pneumonia confirmed by Real-Time Polymerase Chain Reaction for SARS-CoV2 (RT-PCR SARS-CoV2), Berlin protocol, who required hospitalization in three hospitals in Antioquia, Colombia. Patients were treated according to the institutional protocol (from March 20, 2020 to June 30, 2020) with corticosteroid if the patient required supplemental oxygen. From July 1, 2020, the management protocol changed with the addition of colchicine to all patients admitted to the institutions. The treatment was supervised and monitored by the same specialist in Infectology of the institutions. We describe the clinical manifestations and outcomes of the patients who received these treatments. The information of the patients was analyzed according to the outcome of interest (alive/dead) with univariate, bivariate, and multivariate measures to adjust the variables that presented statistical association.

**Results:**

All patients had pneumonia documented by chest computed tomography with ground glass images and presented an alveolar pressure/inspired oxygen fraction (PaFi) less than 300. Three hundred one patients were included, 240 (79.7%) received corticosteroids, within these 145 (48.2%) received colchicine also, and the remaining 61 (20.3%) patients did not receive corticosterioids or colchicine. Mortality in the group that received colchicine was lower compared to the group that did not receive it (9.6 vs 14.6%, p-value = 0.179).

**Conclusions:**

Treatment with corticosteroids and colchicine for managing patients with severe Covid-19 Pneumonia was associated with low mortality at the hospital level. Randomized, placebo-controlled studies are required to evaluate the effect of corticosteroids and colchicine on complications or death from Covid-19.

## Introduction

Coronavirus disease 2019 (Covid-19) is caused by the virus classified as Severe Acute Respiratory Syndrome Coronavirus 2 (SARS-CoV-2), an emerging pathogen initially identified in Wuhan-China, in December 2019 [1]. Until August 29, 2020, 25,078,215 infected and 844,515 deaths have been identified globally [2] with 5% lethality and greater extension than previous SARS-CoV and MERS-CoV epidemics [3].

In the Americas, as of August 29, 2020, 13,018,693 cases had been reported, 458,628 deaths with a fatality of 3.52% [4]. In Colombia, the first case was reported, on March 6, 2020, in a 19-year-old patient from Milan, Italy [5]. By August 29, 2020, 582,022 cases and 18,468 deaths were confirmed for a fatality of 3.1%. The clinical manifestations are diverse; some patients present pneumonia, with fever, cough, dyspnea, and headache as cardinal symptoms [6], but asymptomatic infections and multi-organ involvement have also been described [7].

To date, there is no antiviral drug treatment or vaccine for the prevention and treatment of Covid-19 [8]. The experience obtained in the pharmacological treatment of the previous SARS-CoV and MERS-CoV epidemics has been extrapolated to the current pandemic, the results of the few studies carried out to date for Covid-19 being controversial. In vitro studies demonstrated antiviral activity against SARS-CoV-2 from Hydroxychloroquine [9] and Lopinavir/Ritonavir [10], although its clinical use did not show a decrease in mortality, as did remdesivir [11], Azithromycin was evaluated in non-randomized clinical studies for Covid-19 [12]. There have also been some experimental studies with different glucocorticoids and biological drugs such as Tocilizumab. However, so far, the only therapy that has been shown to decrease mortality from Covid-19 was dexamethasone [13].

In a small randomized clinical study, Greek researchers evaluated the effect of colchicine on cardiac and inflammatory markers in patients infected with Covid-19. Although they did not find differences in biomarkers concerning standard therapy, they showed less clinical deterioration determined by less mechanical ventilation and deaths in 55 patients who received colchicine than the conventional therapy group [14].

Since there is no specific therapy against SARS-CoV2, current efforts aim to prevent contagion through public health measures and develop a protective vaccine [15]. While waiting for the latter, it is necessary to evaluate the drugs that at least, in initial studies, suggested some degree of utility in the management of Covid-19 or its complications, such as Acute Respiratory Distress Syndrome (ARDS) or cytokine storm. The objective of this study was to describe the clinical manifestations and outcomes of patients with severe Covid-19 Pneumonia treated with corticosteroids and colchicine.

## Materials and methods

An observational study was conducted in three clinics in Antioquia (two in Medellín and one in Apartadó), a department located in the northwest of Colombia. Its capital is Medellin, the second most populated city in Colombia; aside, Apartadó is a municipality located in the Urabá subregion, located 310 km from Medellin, whose hospital is a second-level reference center of the region. The included patients were older than 18 years old, hospitalized for Covid-19 Pneumonia, confirmed positive by Real-Time Reverse Transcription Polymerase Chain Reaction for SARS-CoV2 (RT-PCR SARS-Cov2) by Berlin protocol. The samples were taken from a nasopharyngeal swab. The patients should also have radiological confirmation of Pneumonia, mostly chest tomography or chest X-rays, to support the diagnosis of Covid-19 Pneumonia.

In total, 301 patients met the inclusion criteria. After obtaining informed consent, patients were treated according to the institutional protocol (from March 20, 2020 to June 30, 2020) with corticosteroid if the patient required supplemental oxygen. From July 1, 2020, the management protocol changed with the addition of colchicine to all patients admitted to the institutions. The treatment was supervised and monitored by the same specialist in Infectology of the institutions. Corticosteroid treatment was mostly with dexamethasone, some with prednisolone or methylprednisolone, and colchicine at a dose of 0.5 mg every 12 h for 7 to 14 days.

Upon admission, a blood count, kidney and liver function tests, arterial gases, lactate dehydrogenase, D-dimer, serum ferritin, and C-reactive protein were performed. Low molecular weight heparins were prescribed to all patients to prevent thromboembolism during their hospital stay and pronation according to tolerance if arterial oxygen pressure/expired fraction of oxygen (PaFi) less than 300.

Pneumonia was classified as mild if the patients did not have hypoxemia or need for supplemental oxygen; Severe pneumonia was defined by the presence of hypoxemia or supplemental oxygen requirement, septic shock syndrome, or multisystem compromise. The Acute Respiratory Distress Syndrome (ARDS) was defined as the presence of bilateral pulmonary infiltrates not explained by another etiology and Covid-19 and PaFi less than 300. Standardization was carried out under the observation of the researcher, thus guaranteeing adequate techniques in collecting information. With these data, a database was built in Microsoft Excel, and before the analysis, it was subjected to quality control.

The variable of interest was the outcome (dead (n = 37)/alive (n = 264)) and the factors analyzed were: demographic (age and sex), comorbidities (high blood pressure, diabetes mellitus, obesity, dementia, cancer, hypothyroidism, kidney failure, coronary heart disease, chronic obstructive pulmonary disease (COPD), asthma, dyslipidemia, autoimmune disease, psychiatric disease, heart failure) risk factor like smoking or tobacco addiction, clinical manifestations dyspnea, cough, fever, chest pain, asthenia, anosmia, diarrhea, headache, odynophagia; hospital care and admission to the intensive care unit, type of supplemental oxygen requirement, nasal cannula, non-invasive ventilation, high-flow cannula, ventury oxygen or non-rebreathing mask, mechanical ventilation, treatment received: corticosteroid, colchicine, findings laboratory tests: lymphocytes, lactic dehydrogenase, serum ferritin, D-dimer, baseline and lowest PaFi during hospitalization.

Once all the variables had been collected, proceed to their univariate analysis with the calculation of frequencies and statistics, bivariate analysis with the outcome variable, and the chi-square hypothesis tests were calculated, with statistical significance (p-value < 0.05). As an epidemiological measure, the crude and adjusted OR were calculated, with their confidence intervals (95% CI); For the multivariate analysis, binary logistic regression was used, and the variables that met the Hosmer–Lemeshow criteria were entered into the final model (p-value < 0.25). All calculations were made with the SPSS version 21 package (CES University license).

### Ethical considerations

The study was approved by the ethics committees of Clínica Medellín, Nueva Clínica Sagrado Corazón, and Clínica Panamericana. Informed consent was obtained from the study participants.

## Results

**Fig. 1:**
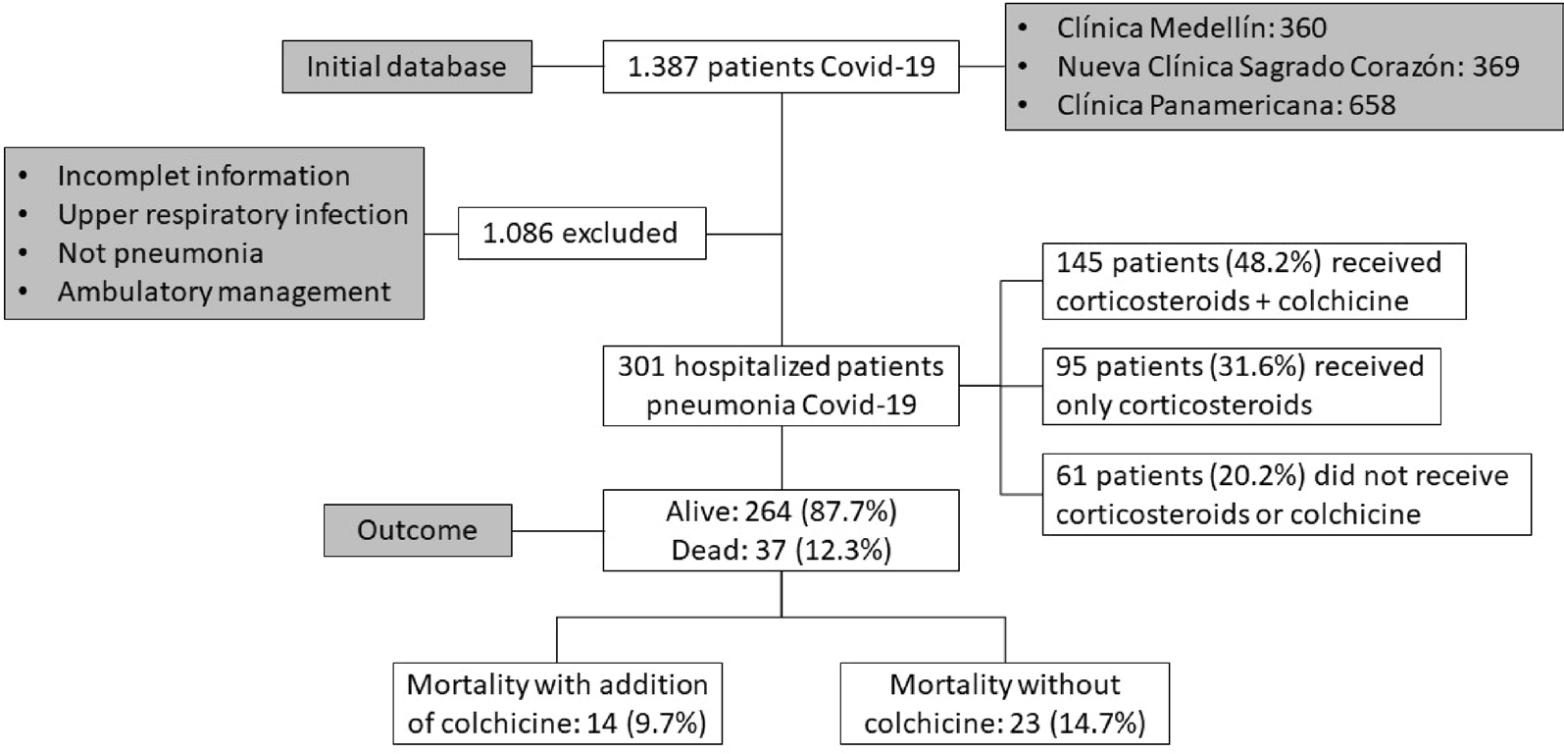
Patients with Covid-19 infection in three clinics in Antioquia, Colombia, between March 20 and August 7, 2020

In the period between March 20, 2020, and August 7, 2020, 1,387 patients with Covid-19 infection confirmed by RT-PCR SARS Cov2, Berlin protocol, were diagnosed from a sample taken from the nasopharyngeal swab, in 3 clinics of Antioquia, 360 at Clínica Medellín (Medellín, Colombia), 369 at Nueva Clinica Sagrado Corazón (Medellín, Colombia) and 658 at Clinica Panamericana (Apartadó, Colombia). One thousand eighty-six patients were discarded because they did not have imaging findings on chest radiography or chest computed tomography compatible with Pneumonia, due to incomplete information or discharge from the emergency room for outpatient management (Fig. 1).

**Fig. 2:**
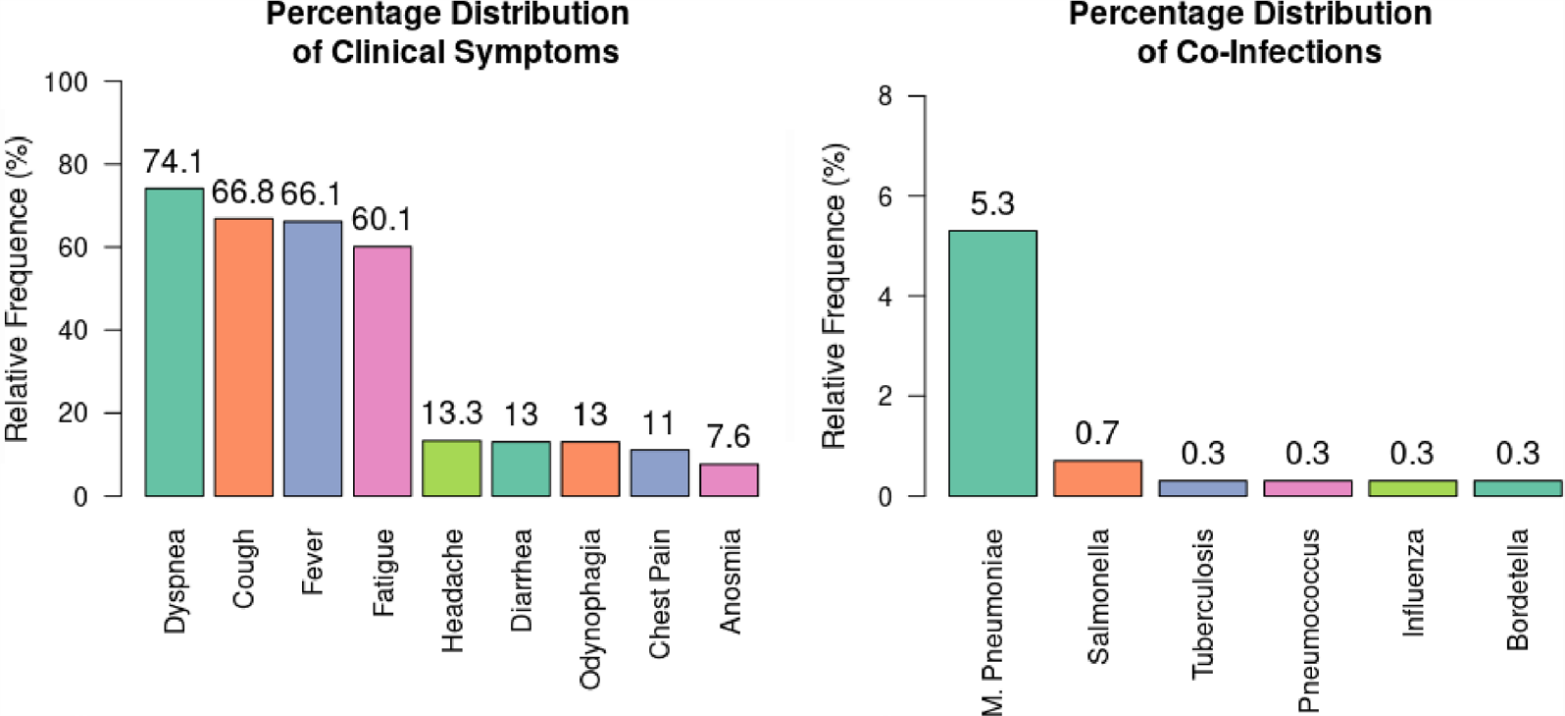
Proportional distribution of hospitalized patients with Covid-19 Pneumonia, according to clinical manifestations and presence of coinfection

Of the 301 patients included for the analysis, one hundred seventy eight (59.1%) corresponded to male patients, and the average age was 56.8 years (SD 17.34 years). Two hundred twenty five (74.8%) presented some comorbidity, of which the most frequent were arterial hypertension, one hundred and thirty seven (45.5%), and diabetes mellitus 73 (24.3%), forty two (13.9%) were obese. Table 1. Most patients had symptoms such as dyspnea (74.1%), cough (66.8%), and fever (66.1%). Twenty three (7.6%) had a coinfection on admission; the most frequent were Mycoplasma pneumoniae infection (Fig. 2).

**Table 1:** Frequency distribution of patients hospitalized for Covid-19 Pneumonia according to demographic characteristics and comorbidities

Variable	Treatment			Total patients (n = 301)	Pearson’s Chi-Squared p-value
	Corticosteroids (n = 95)	Corticosteroids AndColchicine (n = 145)	Without Corticosteroids (n = 61)		
Age	55.9 (± 18)	59.1 (± 16.5)	53.7 (± 17.5)	56.8 (± 17.3)	–
Sex					
Male	61 (64.2%)	75 (51.7%)	42 (68.9%)	178 (59.1%)	0.04
Female	34 (35.8%)	70 (48.3%)	19 (31.1%)	123 (40.9%)	
Comorbidities	71 (74.7%)	109 (75.2%)	45 (73.8%)	225 (74.8%)	0.98
Cancer	5 (5.3%)	9 (6.2%)	2 (3.3%)	16 (5.3%)	0.69
Hypothyroidism	4 (4.2%)	18 (12.4%)	7 (11.5%)	29 (9.6%)	0.09
Heart disease	1 (1%)	5 (3.5%)	1 (1.9%)	7 (2.3%)	0.45
Renal failure	2 (2.1%)	12 (8.3%)	4 (6.6%)	18 (5.9%)	0.14
Dementia	2 (2.1%)	6 (4.1%)	4 (6.6%)	12 (3.9%)	0.38
Obesity	22 (23.2%)	15 (10.3%)	5 (8.2%)	42 (13.9%)	0.007
COPD	12 (12.6%)	15 (10.3%)	3 (4.9%)	30 (9.9%)	0.29
Diabetes mellitus	21 (22.1%)	38 (26.2%)	14 (22.9%)	73 (24.3%)	0.74
Hypertension	44 (46.3%)	67 (46.2%)	26 (42.6%)	137 (45.5%)	0.88
Dyslipidemia	4 (4.2%)	19 (13.1%)	4 (6.6%)	27 (8.9%)	0.05
Autoimmune disease	3 (3.2%)	1 (0.7%)	3 (4.9%)	7 (2.3%)	0.15
Psychiatric disease	1 (1.1%)	5 (3.4%)	1 (1.6%)	7 (2.3%)	0.45
Neurological disease	6 (6.3%)	1 (0.7%)	4 (6.6%)	11 (3.7%)	0.03
Coronary disease	5 (5.3%)	11 (7.6%)	2 (3.3%)	18 (5.9%)	0.46
Tobacco addiction	11 (11.6%)	14 (9.7%)	9 (14.8%)	34 (11.3%)	0.57

According to the pharmacological treatment, only 17 (5.6%) received Hydroxychloroquine and lopinavir/ritonavir. Antibiotics were prescribed to 272 (90.4%) of the patients, the most commonly used being ceftriaxone in 205 (68.1%), and azithromycin 219 (72.8%). By institutional protocol, antibiotics were administered but no coinfection was documented in most cases, for which they had no effect on the clinical outcome. Two hundred forty patients (79.7%) received corticosteroids, mainly dexamethasone in 227 (93.4%), and 145 (48.2%) received both corticosteroids and colchicine. One hundred and five patients (34.9%) were admitted to the intensive care unit, but only 57 (18.9%) need mechanical ventilation. Table 2.

**Table 2:** Proportional distribution of hospitalized patients with Covid-19 Pneumonia according to the treatment received

Variables	Treatment			Total patients (n = 301)	Pearson’s Chi-Squared p-value
	Corticosteroids (n = 95)	Corticosteroids AndColchicine (n = 145)	Without Corticosteroids (n = 61)		
Hydroxychloroquine and Lopinavir/Ritonavir	8 (8.4%)	0 (0%)	9 (14.8%)	17 (5.6%)	< 0.001
Other Antibiotics	95 (100%)	137 (94.5%)	40 (65.8%)	272 (90.4%)	< 0.001
Ceftriaxone	64 (67.4%)	112 (77.2%)	29 (47.5%)	205 (68.1%)	0.002
Azithromycin	75 (78.9%)	122 (84.1%)	22 (36.1%)	219 (72.8%)	< 0.001
Tocilizumab	0 (0%)	1 (0.7%)	0 (0%)	1 (0.3%)	0.58
Dexamethasone	91 (95.8%)	133 (91.7%)	0 (0%)	224 (74.4%)	< 0.001
ICU Admission	45 (47.4%)	48 (33.1%)	12 (19.8%)	105 (34.9%)	0.002
No Data	1 (1.1%)	2 (1.4%)	0 (0%)	3 (0.9%)	
ConventionalOxygen therapy	91 (95.8%)	129 (88.9%)	45 (73.8%)	265 (88%)	0.002
No data	0 (0%)	2 (1.4%)	0 (0%)	2 (0.7%)	
High-flow nasal canula	62 (65.3%)	62 (42.8%)	12 (19.7%)	136 (45.2%)	< 0.001
No data	1 (1.1%)	3 (2.1%)	0 (0%)	4 (1.3%)	
Mechanical ventilation	28 (29.5%)	26 (17.9%)	3 (4.9%)	57 (18.9%)	< 0.001
No data	1 (1.1%)	2 (1.4%)	0 (0%)	3 (0.9%)	

The patients who died presented as complications ARDS (94.6%), shock (64.9%), Renal Injury (48.6%), and secondary infection (27%). Regarding the patients admitted to the ICU, 59.5% died. Colchicine was administered in 145 patients and of them 14 (9.7%) died vs 23 (14.8%) of those who did not receive it, presenting a non-significant statistical association (p-value = 0.179), but a reduction in fatal outcome was evidenced by 38.2% (OR = 0.618; 95% CI: 0.305–1.253). Table 3. The variables with a statistical association, associated with death were: comorbidity, dementia, kidney injury (renal failure), secondary infection, cancer, co-infection, ARDS, ICU admission, high-flow nasal canula and mechanical ventilation.

**Table 3:** Frequency distribution of patients hospitalized for Covid-19 Pneumonia, according to the outcome (dead/alive) and its associated factors

Variables	Outcome		Wald test p-value	OR	95% confidence interval		Adjusted OR	95% confidence interval	
	Death (n = 37)	Alive (n = 264)			Lower	Upper		Lower	Upper
Colchicine	14 (37.8%)	131 (0.5%)	0.18	0.618	0.305	1.253	0.899	0.211	3.83
Comorbidities	34 (91.9%)	191 (72.3%)	0.018	4.332	1.291	14.539	4.827	0.324	71.83
Hypertension	20 (54.1%)	117 (44.3%)	0.267	1.478	0.741	2.949	0.624	0.065	6.02
Diabetes Mellitus	10 (27%)	63 (23.9%)	0.674	1.182	0.542	2.575	5.909	0.609	57.361
Obesity	7 (18.9%)	35 (13.3%)	0.355	1.527	0.623	3.741	0.508	0.061	4.267
Hypothyroidism	5 (13.5%)	24 (9.1%)	0.4	1.56	0.56	4.38	0.247	0.017	3.559
Renal Failure/Injury	18 (48.6%)	36 (13.6%)	< 0.001	6	2.879	12.504	12.452	1.301	119.165
Coronary Disease	4 (10.8%)	13 (4.9%)	0.2	2.17	0.67	6.97	15.932	0.721	352.075
COPD	5 (13.5%)	25 (9.5%)	0.44	1.49	0.53	4.18	1.013	0.167	6.145
Dementia	4 (10.8%)	8 (3%)	0.034	3.879	1.107	13.59	294.178	4.054	21,349.556
Cancer	7 (18.9%)	9 (3.4%)	< 0.001	6.611	2.296	19.038	589.909	17.691	19,670.832
Dyslipidemia	2 (5.4%)	25 (9.5%)	0.42	0.55	0.12	2.41	1.394	0.075	25.897
Autoimmune Disease	2 (5.4%)	5 (1.9%)	0.21	2.96	0.55	15.84	25.119	0.494	1276.397
Psychiatric Disease	1 (2.7%)	6 (2.3%)	0.87	1.19	0.14	10.21	3.291	0.039	280.52
Neurological Disease	2 (5.4%)	9 (3.4%)	0.55	1.62	0.34	7.8	0.362	0.001	99.943
Heart Disease	1 (2.7%)	6 (2.3%)	0.87	1.19	0.14	10.21	5.493	0	65,179.579
Secondary Infection	10 (27%)	16 (6.1%)	< 0.001	5.741	2.371	13.9	0.097	0.007	1.289
Shock	24 (64.9%)	18 (6.8%)	< 0.001	25.23 1	11.031	57.712	1802.79 9	16.195	200,680.618
Co-Infection	7 (18.9%)	16 (6.1%)	0.009	3.617	1.377	9.499	15.925	0.845	300.105
ARDS	35 (94.6%)	180 (68.2%)	0.004	8.167	1.919	34.756	0.174	0.002	13.756
Mild (n = 236)	2 (5.4%)	70 (26.5%)	0.003	9.154	2.14	39.24	0.158	0	28,166,402.9 5
Moderate (n = 236)	10 (27%)	62 (23.5%)	0.7	1.168	0.531	2.57	0.005	0	73.57
Severe (n = 237)	25 (67.6%)	67 (25.4%)	< 0.001	4.545	2.11	9.792	1645.53 4	0.091	29,789,951.2 9
Mechanical Ventilation	20 (54.1%)	37 (14%)	< 0.001	8.144	3.83	17.316	0.178	0.003	10.675
High-FlowNasal Canula	32 (86.5%)	104 (39.4%)	0.03	0.083	0.0284	0.241	0.069	0.0038	0.63
ICU admission	22 (59.5%)	83 (31.4%)	0.001	3.389	1.651	6.954	1.213	0.099	14.824
Lymphocytes									
(900,3300)	13 (35.1%)	138 (52.3%)	0.054	2.022	0.987	4.141	0.065	0.006	0.759
< 900 or > 3300	24 (64.9%)	126 (47.7%)							
Ferritin									
< 204	2 (5.4%)	31 (11.7%)	0.261	2.328	0.534	10.161	0.059	0.003	1.292
> 204	35 (94.6%)	233 (88.3%)							
LDH									
< 220	0 (0%)	36 (13.6%)	0.998	NA	–	–	–	–	–
> 220	37 (100%)	228 (86.4%)							
D-Dimer									
< 198	1 (2.7%)	19 (7.2%)	0.324	0.358	0.047	2.758	1.084	0	298,440.882
> 198	36 (97.3%)	245 (92.8%)							
Initial PaFi									
< 200	28 (75.7%)	95 (36%)	< 0.001	5.535	2.507	12.219	0.472	0.053	4.228
> 200	9 (24.3%)	169 (64%)							
< 300	34 (91.9%)	190 (72%)	0.016	4.414	1.315	14.812	2.57	0.094	70.038
> 300	3 (8.1%)	74 (28%)							
Lowest PaFi									
< 200	35 (94.6%)	140 (53%)	< 0.001	15.5	3.653	65.769	1.399	0	1,870,118,737
> 200	2 (5.4%)	124 (47%)							
< 300	37 (100%)	221 (83.7%)	0.997	NA	–	–	–	–	–

We perform a variable selection procedure in the binary logistic regression, specifically, the backward selection method, which performs iteratively multiple logistic regressions and, in each iteration it is removed the explanatory variable that is not (and also less) significant in the resultant model. Thereby, the significant variables belongs to a full significant model with a Nagelkerke R2 score of 0.745. Table 4. All the variables that met the Hosmer–Lemeshow criteria and the included by medical criteria were entered-documenting an increase in the probability of dying in patients with Covid-19 Pneumonia who had some comorbidity, dementia, cancer, kidney injury, co-infection, and any degree of ARDS, which required admission to the ICU and those who had PaFi less than 200.

**Table 4:** Full significant logistic model with Nagelkerke R2 score of 0.745, according to the outcome (dead/alive) and its associated factors

Variables	Outcome		Wald test p-value	OR	95% confidence interval		Adjusted OR	95% confidence interval	
	Death (n = 37)	Alive (n = 264)			Lower	Upper		Lower	Upper
Comorbidities	34 (91.9%)	191 (72.3%)	0.018	4.332	1.291	14.539	4.827	0.324	71.83
Renal Failure/Injury	18 (48.6%)	36 (13.6%)	< 0.001	6	2.879	12.504	12.452	1.301	119.165
Dementia	4 (10.8%)	8 (3%)	0.034	3.879	1.107	13.59	294.17 8	4.054	21,349.556
Cancer	7 (18.9%)	9 (3.4%)	< 0.001	6.611	2.296	19.038	589.90 9	17.691	19,670.832
Secondary Infection	10 (27%)	16 (6.1%)	< 0.001	5.741	2.371	13.9	0.097	0.007	1.289
Shock	24 (64.9%)	18 (6.8%)	< 0.001	25.23 1	11.031	57.712	1802.7 99	16.195	200,680.618
Co-Infection	7 (18.9%)	16 (6.1%)	0.009	3.617	1.377	9.499	15.925	0.845	300.105
ARDS	35 (94.6%)	180 (68.2%)	0.004	8.167	1.919	34.756	0.174	0.002	13.756
Mild (n = 236)	2 (5.4%)	70 (26.5%)	0.003	9.154	2.14	39.24	0.158	0	28,166,402.95
Severe (n = 237)	25 (67.6%)	67 (25.4%)	< 0.001	4.545	2.11	9.792	1645.5 34	0.091	29,789,951.29
Mechanical Ventilation	20 (54.1%)	37 (14%)	< 0.001	8.144	3.83	17.316	0.178	0.003	10.675
High-FlowNasal Canula	32 (86.5%)	104 (39.4%)	0.03	0.083	0.0284	0.241	0.069	0.0038	0.63
ICU admission	22 (59.5%)	83 (31.4%)	0.001	3.389	1.651	6.954	1.213	0.099	14.824
Initial PaFi									
< 200	28 (75.7%)	95 (36%)	< 0.001	5.535	2.507	12.219	0.472	0.053	4.228
> 200	9 (24.3%)	169 (64%)							
< 300	34 (91.9%)	190 (72%)	0.016	4.414	1.315	14.812	2.57	0.094	70.038
> 300	3 (8.1%)	74 (28%)							
Lowest PaFi									
< 200	35 (94.6%)	140 (53%)	< 0.001	15.5	3.653	65.769	1.399	0	1,870,118,737
> 200	2 (5.4%)	124 (47%)							

**Fig. 3:**
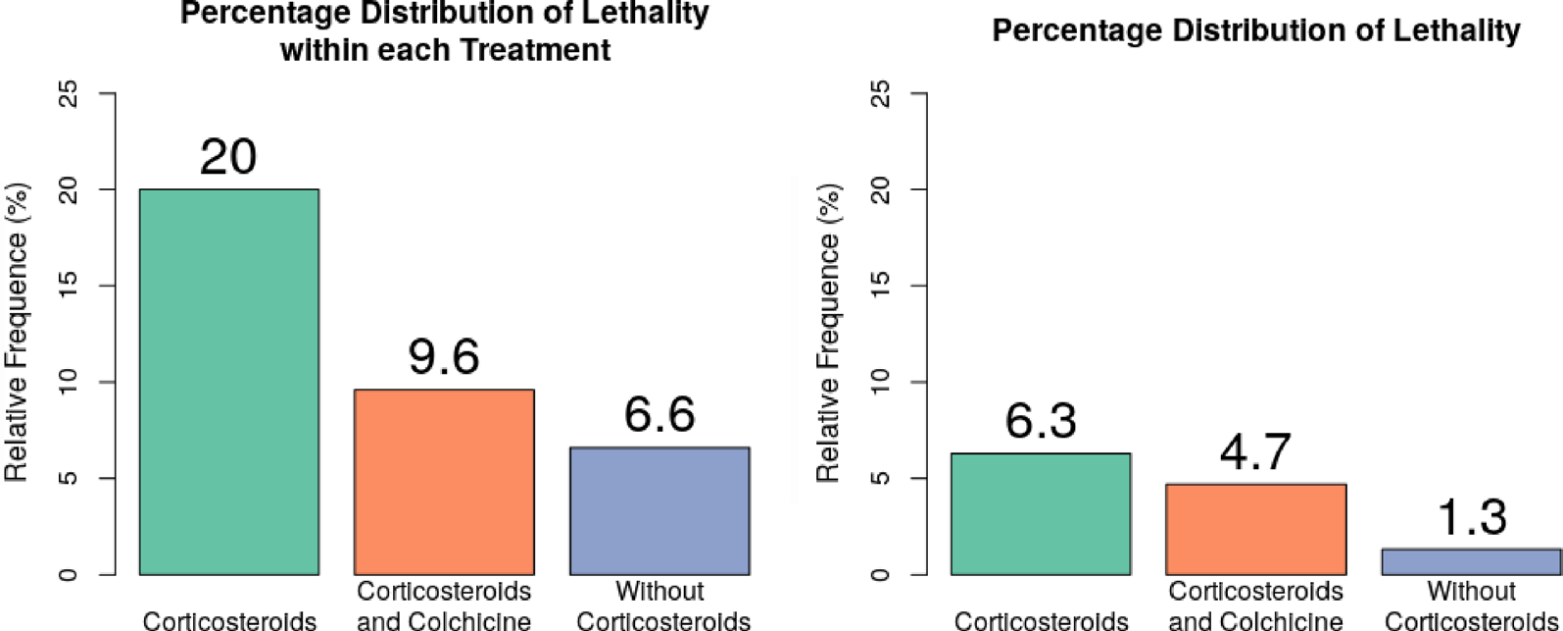
Percentage distribution of lethality

Hospital mortality from severe Covid-19 Pneumonia was 12.3% in total, i.e. considering all 301 patients, where people that received corticosteroids and colchicine had less lethality percentage (4.7 vs 6.3%). 54.1% of the patients who required mechanical ventilation died. Lethality within each treatment group was also compared, patients in the group that received corticosteroids and colchicine had less mortality than patients that received only corticosteroids (9.6 vs 20%). Figure 3. left panel. Less lethality in the without-corticosteroids treatment is due to the fact that those patients, at the beginning, did not required supplemental oxygen, therefore, their health was not significantly compromised (6.6 and 1.3%) (Fig. 3).

## Discussion

To date, there is no specific treatment for Covid-19 infection, and in some countries, drugs used in previous coronavirus outbreaks (SARS-coronavirus and MERS-coronavirus) are administered despite little or no evidence of their effectiveness. For SARS-CoV2. The first experience of managing patients with drugs with antiviral activity such as Interferon α, Lopinavir/Ritonavir, Chloroquine, Ribavirin, and Umifenovir (the latter not available in Colombia) was proposed in the guide for the prevention, diagnosis, and treatment of pneumonia due to Covid- 19 from the ROC National Health Commission (6th version, released February 18, 2020) [16]. Lopinavir/Ritonavir showed some activity against SARS-CoV and MERS-CoV. However, in a recent study in patients with SARS-CoV2, the use of Lopinavir/Ritonavir 400/100 mg every 12 h for 14 days did not demonstrate statistically significant differences in mortality compared to standard treatment without drug therapy (19.2% vs. 25.0%; 95% CI, − 17.3 to 5.7). In vitro, Hydroxychloroquine was more potent antiviral than Chloroquine, making Hydroxychloroquine a possible therapeutic option [17], thanks to the inhibition of viral replication due to its immunomodulatory effects [9]. However, the study by Geleris et al. [18] in 1376 patients, of whom 811 (58.9%) received Hydroxychloroquine, there were no differences regarding intubation or death compared to the group that did not receive it (hazard ratio, 1.04, 95% confidence interval, 0.82 to 1.32).

Although the administration of corticosteroids was controversial at the beginning of the pandemic, given the clinical studies in other viral respiratory infections (respiratory syncytial virus, influenza, SARS-coronavirus or MERS-coronavirus) that associated the use of corticosteroids with increased mortality and infections nosocomial, higher viral persistence and a higher rate of adverse reactions such as psychosis (dose-dependent), diabetes and vascular necrosis [19]. Recent evidence changed opinion about them Villar et al. [20], in a placebo-controlled, randomized, and multicenter study, found that patients with severe acute respiratory distress syndrome (ARDS) treated with dexamethasone had lower mortality (21 vs. 36%, p-value < 0.0047) and Wu et al. [21] in patients with ARDS due to Covid-19, showed a decrease in the risk of death in patients treated with methylprednisolone (HR, 0.38; 95% CI, 0.20–0.72). More recently, RECOVERY study [13] found that patients who received dexamethasone had a decrease in mortality in a third of ventilated patients (frequency ratio 0.65 [95% confidence interval: 0.48 to 0.88]; p-value = 0.0003) and in a fifth in other patients who received oxygen only [0.80 (0.67–0.96); p-value = 0.0021].

Different studies have documented a cytokine release syndrome in patients with severe Covid19 [22], with an increase in tumor necrosis factor-α (TNF-α), followed by an increase in Interleukin (IL) -1β, IL-2, IL-6, IL-8, IL-10, and interferon γ (IFN-γ) [23]. Colchicine, an alkaloid derivative of the Colchicum genus plants, inhibits IL-1β and IL-18 by interacting with the inflammasome Nod-like receptor protein 3 inflammasome protein complex 1 (NLRP-3), for which it is hypothesized that it could be useful in severe Covid-19 Pneumonia [24].

Mansouri et al. [25] described the improvement of a 42-year-old patient with Covid-19 Pneumonia and cytokine release syndrome with early administration of colchicine, evidenced by clinical improvement and decrease in severity markers, including ferritin, D dimer, and normalization of levels of IL-6. Scarsi et al. [26] conducted a proof-of-concept study in a single hospital in Italy, where they administered colchicine to 122 hospitalized patients with Covid-19 Pneumonia. They compared them with a standard care group without colchicine, showing a lower risk in the survival analysis of death in those patients who received colchicine [HR = 0.151 (95% CI 0.062–0.368)].

In the GRECCO study [14], the use of colchicine decreased the clinical deterioration of patients compared to patients who did not receive it (1.8 vs 14%, OR 0.11; 95% CI, 0.01–0.96; p-value = 0.02). According to our literature review, this is the first multicenter study reported to date, with a greater number of patients who were administered corticosteroids plus colchicine (145) for the management of Covid-19 Pneumonia. 14 (9.7%) died vs 23 (14.7%) of those who did not receive it (p-value = 0.179), but there was a decrease in fatal outcome by 38.2% (OR = 0.618; 95% CI: 0.305–1.253). Factors that possibly affected the result can be explained by the sample's size, mainly, and to a lesser extent, the time of initiation of the drug.

Regarding mortality in our population, patients with Covid-19 Pneumonia who received management with corticosteroids and colchicine had an overall mortality of 12.3%, low compared to hospital statistics from developed countries, such as Germany, which has been recognized as one of the few countries that did not see its capacity to respond to the pandemic exceeded. In an observational study by Karagiannidis et al. [27] in 10,021 patients in 920 hospitals in the German country, the overall mortality was 22%, 16% for those who did not require mechanical ventilation vs. 53% for those who did. Due to the observational nature of this study, the reported findings should be interpreted with caution. Randomized, placebo-controlled clinical studies are required to evaluate the effect of administered drug therapy.

## Conclusions

Treatment with corticosteroids and colchicine for managing patients with severe Covid-19 Pneumonia was associated with low mortality at the hospital level. Randomized, placebo-controlled studies are required to evaluate the effect of this therapy.

## Data Availability

The datasets of the current study are available from the corresponding author on reasonable request.

## References

[1] Zhu N, Zhang D, Wang W (2020). A novel coronavirus from patients with pneumonia in China, 2019. N Engl J Med.

[2] Coronavirus Update (Live): 30,157,437 Cases and 947,034 deaths from COVID-19 virus pandemic—worldometer [Internet]. Worldometers.info. 2020 [cited 29 August 2020]. https://www.worldometers.info/coronavirus/?zarsrc=130.

[3] Mahase E (2020). Coronavirus COVID-19 has killed more people than SARS and MERS combined, despite lower case fatality rate. BMJ.

[4] Pan American Health Organization, World Health Organization. COVID-19 information system for the region of the Americas, as of 29 Aug 2020. https://paho-COVID19-response-who.hub.arcgis.com/. Accessed 29 Aug 2020.

[5] Ministerio de Salud y Protección Social. Colombia confirma su primer caso de COVID-19. Colombia M. Colombia confirma su primer caso de COVID-19 [Internet]. Minsalud.gov.co. 2020 [cited 29 August 2020]. https://www.minsalud.gov.co/Paginas/Colombia-confirma-su-primer-caso-de-COVID-19.aspx.

[6] Lupia T, Scabini S, Mornese Pinna S (2020). 2019 novel coronavirus (2019-nCoV) outbreak: a new challenge. J Glob Antimicrob Resist.

[7] COVID-19 guideline, part 1: treatment and management [Internet]. Idsociety.org. 2020 [cited 29 August 2020]. https://www.idsociety.org/practice-guideline/covid-19-guideline-treatment-and-management/

[8] Infectious Diseases Society of America Guidelines on the Treatment and Management of Patients with COVID-19. Last updated April 13, 2020 at 4:39 PM EDT and posted online at www.idsociety.org/COVID19guidelines 10.1093/cid/ciaa478 PMC7197612 32338708

[9] Yao X, Ye F, Zhang M (2020). In vitro antiviral activity and projection of optimized dosing design of hydroxychloroquine for the treatment of severe acute respiratory syndrome coronavirus 2 (SARS-CoV-2). Clin Infect Dis..

[10] Yao TT, Qian JD, Zhu WY, Wang Y, Wang GQ (2020). A systematic review of lopinavir therapy for SARS coronavirus and MERS coronavirus-A possible reference for coronavirus disease-19 treatment option. J Med Virol..

[11] de Wit E, Feldmann F, Cronin J (2020). Prophylactic and therapeutic remdesivir (GS-5734) treatment in the rhesus macaque model of MERS-CoV infection. Proc Natl Acad Sci U S A.

[12] Gautret P, Lagier J-C, Parola P (2020). Hydroxychloroquine and azithromycin as a treatment of COVID-19: results of an open-label non-randomized clinical trial. Int J Antimicrob Agents.

[13] RECOVERY Collaborative Group, Horby P, Lim WS, Emberson JR, Mafham M, Bell JL, et al Dexamethasone in hospitalized patients with Covid-19—preliminary report. N Engl J Med. 2020; 17:NEJMoa2021436. doi: 10.1056/NEJMoa2021436.

[14] Deftereos S, Giannopoulos G, Vrachatis D, Siasos GD, Giotaki S, Gargalianos P (2020). Effect of colchicine vs standard care on cardiac and inflammatory biomarkers and clinical outcomes in patients hospitalized with coronavirus disease 2019: the GRECCO-19 randomized clinical trial. JAMA Netw Open..

[15] Dong L, Hu S, Gao J (2020). Discovering drugs to treat coronavirus disease 2019 (COVID-19). Drug Discov Ther.

[16] Jin YH, Cai L, Cheng ZS (2020). A rapid advice guideline for the diagnosis and treatment of 2019 novel coronavirus (2019-nCoV) infected pneumonia (standard version). Mil Med Res.

[17] Colson P, Rolain J, Lagier J, Brouqui P, Raoult D (2020). Chloroquine and hydroxychloroquine as available weapons to fight COVID-19. Int J Antimicrob Agents.

[18] Geleris J, Sun Y, Platt J (2020). Observational study of hydroxychloroquine in hospitalized patients with Covid-19. N Engl J Med.

[19] Russell CD, Millar JE, Baillie JK (2020). Clinical evidence does not support corticosteroid treatment for 2019-nCoV lung injury. Lancet.

[20] Jesús V, Carlos F, Domingo M (2020). Dexamethasone treatment for the acute respiratory distress syndrome: a multicenter, randomised controlled trial. Lancet Respir Med.

[21] Chaomin Wu, Xiaoyan C, Yanping C (2020). Risk factors associated with acute respiratory distress syndrome and death in patients with coronavirus disease 2019 pneumonia in Wuhan, China. JAMA Intern Med.

[22] Lee DW, Gardner R, Porter DL (2014). Current concepts in the diagnosis and management of cytokine release syndrome. Blood.

[23] Mehta P, McAuley DF, Brown M, Sanchez E, Tattersall RS, Manson JJ (2020). COVID-19: consider cytokine storm syndromes and immunosuppression. Lancet.

[24] Martinon F, Pétrilli V, Mayor A, Tardivel A, Tschopp J (2006). Gout associated uric acid crystals activate the NALP3 inflammasome. Nature.

[25] Mansouri N, Marjani M, Tabarsi P, von Garnier C, Mansouri D (2020). Successful Treatment of Covid-19 Associated Cytokine Release Syndrome with Colchicine. A case report and review of literature. Immunol Invest..

[26] Scarsi M, Piantoni S, Colombo E, Airó P, Richini D, Miclini M (2020). Association between treatment with colchicine and improved survival in a single-centre cohort of adult hospitalised patients with COVID-19 Pneumonia and acute respiratory distress syndrome. Ann Rheum Dis.

[27] Karagiannidis C, Mostert C, Hentschker C (2020). Case characteristics, resource use, and outcomes of 10 021 patients with COVID-19 admitted to 920 German hospitals: an observational study. Lancet Respir Med.

